# Association of TyG-related indices with incident DM among NAFLD patients: a retrospective study

**DOI:** 10.1007/s42000-025-00710-x

**Published:** 2025-08-06

**Authors:** Yuanyang He, Luyao Fu, Jie Gui, Shishi Li, Yun Ai

**Affiliations:** 1 Department of Hepatobiliary Surgery, The Third Hospital of Mianyang, Sichuan Mental Health Center, No.190 Jiannan Road East Section, Youxian District, Mianyang City, 621000 Sichuan People’s Republic of China; 2 Department of Pathology, Luzhou People’s Hospital, Luzhou, Sichuan China; 3 Department of Hepatobiliary Surgery, The First Affiliated Hospital of Chongqing Medical University, Chongqing, China; 4 Department of Urology, The First Affiliated Hospital of Chongqing Medical University, Chongqing, China

**Keywords:** Diabetes mellitus, Triglyceride glucose index, Triglyceride glucose-related indices, Retrospective study, Non-alcoholic fatty liver disease

## Abstract

**Background:**

The purpose of this study was to evaluate the relationship between triglyceride glucose (TyG)-related indices, including the TyG index and its modified versions, and the risk of developing diabetes mellitus (DM) in patients with non-alcoholic fatty liver disease (NAFLD).

**Methods:**

Data were sourced from the Dryad repository, comprising 2,374 individuals diagnosed with NAFLD. The TyG index was calculated using the formula: Ln [(fasting triglyceride level in mg/dL) × (fasting blood glucose in mg/dL)]/2. Modified indices were created by incorporating the TyG index with body mass index (BMI), waist circumference (WC), and waist-to-height ratio (WHtR). Logistic regression analysis and restricted cubic spline regression were utilized to investigate the association of the TyG index and modified indices with the incidence of diabetes. Predictive capabilities were evaluated through receiver operating characteristic (ROC) curve analyses.

**Results:**

In multivariable logistic regression models that accounted for confounding factors, a linear correlation was identified between the TyG index and its related indices (e.g., TyG-BMI, TyG-WC, and TyG-WHtR) with the development of DM, when considered as continuous variables. Each standard deviation (SD) increase was associated with odds ratios (ORs) and 95% confidence intervals (CIs) of 1.45 (1.23–1.72, *P* < 0.001), 1.57 (1.33–1.85, *P* < 0.001), 1.81 (1.53–2.14, *P* < 0.001), and 1.75 (1.48–2.06, *P* < 0.001), respectively. The area under the curve (AUC) values for TyG, TyG-BMI, TyG-WC, and TyG-WHtR were 0.626, 0.647, 0.675, and 0.681, respectively, indicating that the TyG-related indices had higher AUC values than TyG alone. Stratified analyses revealed a positive correlation between TyG-related indices and DM among NAFLD patients with various characteristics.

**Conclusions:**

A linear and positive correlation was observed between TyG-related parameters and the risk of incident DM. Among NAFLD patients, the TyG-BMI, TyG-WC, and TyG-WHtR indices were found to be more effective clinical predictors than the TyG index alone for forecasting the onset of DM.

## Introduction

Diabetes mellitus (DM) is a chronic metabolic disorder characterized by elevated blood glucose levels, which can lead to various complications [1]. Among these, incident DM refers to the development of new-onset diabetes. The onset of incident DM is influenced by a complex interplay of genetic, environmental, and lifestyle factors [2]. Non-alcoholic fatty liver disease (NAFLD) is a prevalent condition characterized by the accumulation of fat in the liver in the absence of significant alcohol consumption, which has emerged as a significant public health issue worldwide [3]. NAFLD is closely associated with metabolic syndrome, a cluster of disorders that includes central obesity, dyslipidemia, hypertension, and insulin resistance, significantly increasing the risk of developing DM [4]. Individuals with NAFLD have a substantially higher prevalence of DM compared to the general population. This comorbidity not only worsens the clinical manifestations of NAFLD but also heightens the risk of cardiovascular events and kidney disease. It appears that individuals with NAFLD and DM experience less favorable clinical outcomes than those without diabetes [5]. Therefore, the rapid development of methods to identify DM in individuals with NAFLD is crucial. This would allow for the timely identification of individuals at higher risk for the disease, ultimately playing a vital role in preventing the health complications associated with diabetes.

The triglyceride glucose (TyG) index has emerged as a novel marker for insulin resistance and metabolic abnormalities across various populations [6]. Recent studies have reported associations between the TyG index and conditions such as NAFLD, chronic kidney disease, DM, and even certain types of cancer [7–10]. These findings highlight the potential clinical utility of the TyG index as a versatile marker of health and disease. Along with the TyG index, other modified TyG indices, including TyG-BMI (TyG index to body mass index), TyG-WHtR (TyG index to waist-to-height ratio), and TyG-WC (TyG index to waist circumference), have been studied as reliable markers of metabolic abnormalities [11, 12]. However, further studies are needed to elucidate the precise mechanisms underlying these associations and to establish clinical guidelines for the use of TyG index-related parameters in clinical practice. Despite growing evidence linking the TyG index and its derivatives to diabetes risk in general populations, their specific association with diabetes mellitus among NAFLD patients remains underexplored. Considering the overlapping pathophysiological mechanisms involving insulin resistance and dyslipidemia in both NAFLD and DM, investigating these indices in the context of NAFLD offers an opportunity to elucidate their role as early predictors of incident DM onset.

Therefore, this retrospective study aims to investigate the longitudinal association between TyG-related indices—TyG, TyG-WC, TyG-BMI, and TyG-WHtR—and incident DM among NAFLD patients. By examining these relationships, we aim to enhance risk stratification strategies and develop targeted interventions to improve patient outcomes.

## Methods

### Data source

The current investigation represents a secondary analysis using data from the DATADRYAD database (accessible at http://www.datadryad.org/). The research utilized data shared by Takuro Okamura et al. with the Dryad database [13]. The terms of use for this database permit researchers from various backgrounds to employ the data for additional analysis, thereby supporting a range of research hypotheses and enhancing the applicability of the database. The database includes comprehensive data on the participants, including their age, gender, waist circumference (WC), body weight, body mass index (BMI), systolic blood pressure (SBP), diastolic blood pressure (DBP), alanine aminotransferase (ALT) levels, fasting plasma glucose (FPG), total cholesterol (TC), aspartate transaminase (AST) levels, gamma-glutamyl transferase (GGT) levels, high-density lipoprotein cholesterol (HDL-C) concentration, smoking habits, physical activity, presence of fatty liver, alcohol consumption, Triglyceride (TG) levels, Hemoglobin A1c (HbA1c) measurements, obesity classification, visceral adiposity, patterns of ethanol consumption, DM status, and the length of follow-up observations.

### Study population

The data collected from a research program conducted at a medical institution in Gifu, Japan, formed the basis of the NAFLD in the Gifu Area, Longitudinal Analysis (NAGALA) database [13]. From 2004 to 2015, participants in the NAGALA study underwent physical assessments, with about 60% receiving one or two examinations annually. The research question was updated, leading to the establishment of the following exclusion criteria: (1) Individuals previously diagnosed with DM, using diabetic medications, or presenting with fasting plasma glucose levels of 7.0 mmol/L or higher during the initial assessment. (2) Participants missing essential demographic information such as age and gender, crucial blood tests, or anthropometric measurements. (3) Those not diagnosed with NAFLD at the start of the study. (4) Subjects taking drugs at the time of the baseline examination. The diagnosis of NAFLD was confirmed through the identification of fatty liver via ultrasound, while ruling out pharmaceuticals, viral infections, or ethanol as contributing factors. The definitive identification was based on four distinct echographic features, each assigned a value ranging from 0 to 6, as follows: hepatic luminosity (graded 0–4), renal-hepatic acoustic differentiation (valued 0–4), unclear vascular delineation (marked 0–1), and reduction in ultrasound penetration (rated 0–2). A total score of 2 or higher indicated a diagnosis of NAFLD. Informed consent for the use of data was obtained from all subjects, and the NAGALA study was approved by the Murakami Memorial Hospital Research Ethics Committee. Since this investigation served as a secondary retrospective cohort analysis, separate ethical approval was not necessary. The entire research process adhered to the principles outlined in the Declaration of Helsinki.

### Data collection and measurements

As per previous references, standard self-administered questionnaires captured the subjects’ comprehensive medical backgrounds and lifestyle aspects. The BMI was calculated by dividing an individual’s weight in kilograms by the square of their height in meters. Based on these calculations, participants were categorized into two groups, as follows: non-obese individuals, defined as those with a BMI less than 25 kg/m², and obese individuals, defined as those with a BMI of 25 kg/m² or greater [14]. Participants were categorized as non-smokers, ex-smokers, or active smokers based on their smoking habits at the study’s onset. Exercise habit was defined as participating in any form of physical activity regularly, at least once a week [15]. Blood samples were collected in the morning following an overnight fast of at least 8 h and analyzed using an automated analyzer with standardized protocols. High blood pressure (HBP) classification followed the guidelines established by the Japanese Society of Hypertension for Hypertension Management (JSH 2019), which identifies HBP as an office blood pressure with a systolic blood pressure (SBP) of 140 mmHg or higher and/or a diastolic blood pressure (DBP) of 90 mmHg or higher [16].

### TyG index and modified indices

The concentrations of FPG and triglycerides were analyzed and determined using standard methods. Anthropometric measurements, including height, weight, and WC, which were utilized for final data analysis, were recorded through standardized, self-completed questionnaires. The WHtR was calculated as WC divided by height. The TyG index was computed using the logarithmic formula: TyG index = Ln [(triglycerides in mg/dL) × (glucose in mg/dL) / 2]. Modified versions of the TyG index were derived by multiplying the TyG by BMI for TyG-BMI, by WC for TyG-WC, and by WHtR for TyG-WHtR, respectively.

### Assessment of new-onset DM events

Incident DM was defined as the primary outcome measure in this study. The definition of incident DM referred to a situation where a primary care physician documented a patient’s DM diagnosis for the first time. Baseline prevalent diabetes was characterized by DM cases reported by the primary care physician, the patient themselves, or the date of first use of antidiabetic drugs [17]. Furthermore, the incidence of DM was assessed during follow-up examinations, allowing for the identification of new-onset cases. The evaluation of diabetes as an outcome measure involved rigorous adherence to recognized diagnostic criteria and robust surveillance to capture relevant clinical events accurately.

### Statistical analysis

Variables were categorized as either continuous or categorical. Continuous variables were then assessed for normal distribution. Variables that met the normality criteria were reported as means ± standard deviations (SD) and compared between groups using the Student’s t-test. Conversely, continuous variables that did not exhibit a normal distribution were presented as medians ± interquartile ranges (IQRs) and analyzed for group differences using the Wilcoxon rank-sum test. Categorical variables were summarized as percentages and analyzed using the chi-square test. To estimate the odds ratios (ORs) and corresponding 95% confidence intervals (CI) for the association between DM and the TyG index, as well as its modified indices, both univariate and multivariate logistic regression models were utilized. In these models, the continuous variables TyG and modified TyG indices were categorized into three groups using tertiles and subsequently transformed into categorical variables for logistic regression analysis. We utilized restricted cubic spline function analysis to examine the linear relationship between the TyG index and its modified indices with incident DM. This methodological approach enabled a detailed exploration of potential non-linear associations, thereby enhancing our understanding of the relationship between these indices and the prevalence of diabetes mellitus within our study cohort. Receiver operating characteristic (ROC) curves were analyzed using logistic regression models. The focus was on comparing the areas underneath the related ROC curves, along with their respective 95% CIs, for the TyG and the modified TyG indices’ ROC area under the curve (AUC). Considering potential variations and interactions, subgroup analyses were conducted based on age, sex, hypertension (HBP), and exercise habits, with the ORs in the subgroup analysis being adjusted. We assessed the possibility of unaccounted confounding factors influencing the association between TyG-related indices and the development of incident DM by calculating E-values [18]. The statistical analysis was conducted using R software, specifically version 4.1.0, provided by the R Foundation for Statistical Computing. A two-tailed *P*-value less than 0.05 was considered to indicate a statistically significant difference.

## Results

### Characteristics of the study population

A total of 2,374 patients ((1,896 men and 478 women) diagnosed with NAFLD were included in the final analysis. The average age ± standard deviation (SD) was 45 ± 8.0 years in the non-DM group and 46 ± 8.0 years in the DM group, respectively. Detailed demographic characteristics of the cohort, stratified by their diagnosis of incident DM, are presented in Table 1. Observable differences were noted between participants with and without DM. Specifically, individuals diagnosed with incident DM exhibited higher age, WHtR, ALT levels, AST levels, body weight, BMI, TyG-WHtR index, AST/ALT ratio, TyG index, HbA1c, TyG-BMI index, GGT, WC, TG, TC, FPG, and TyG-WC index. Importantly, discernible differences in physical activity patterns, smoking status, and HDL-C were observed, with DM patients showing a tendency towards lower levels of physical exercise and HDL-C. There is no significant difference in sex (*P* = 0.834), height (*P* = 0.891), and HBP level (*P* = 0.281).

**Table 1:** Patient demographics and baseline characteristics

Characteristic	Incident DM		*p*-value
	Non-DM, *N* = 2,176^1^	DM, *N* = 198^1^	
**Age**	45 ± 8	46 ± 8	0.032^2^
**Sex**			0.834^3^
Female, n (%)	437 (20.1%)	41 (20.7%)	
Male, n (%)	1,739 (79.9%)	157 (79.3%)	
**Smoking status**			< 0.001^3^
Never	1,074 (49.4%)	77 (38.9%)	
Past	540 (24.8%)	44 (22.2%)	
Current	562 (25.8%)	77 (38.9%)	
**Habit of exercise**			0.047^3^
no	1,842 (84.7%)	178 (89.9%)	
yes	334 (15.3%)	20 (10.1%)	
**WC**,** cm**	86 ± 8	90 ± 9	< 0.001^2^
**ALT**,** IU/L**	26 (20, 39)	31 (23, 45)	< 0.001^2^
**AST**,** IU/L**	20 (16, 25)	21 (17, 28)	0.005^2^
**ALT/AST ratio**	1.42 ± 0.45	1.54 ± 0.50	0.002^2^
**Weight**,** kg**	72 ± 11	75 ± 12	< 0.001^2^
**GGT**,** IU/L**	22 (16, 32)	25 (18, 37)	0.015^2^
**HDL-C**,** mmol/L**	1.19 ± 0.29	1.10 ± 0.24	< 0.001^2^
**TC**,** mmol/L**	5.43 ± 0.86	5.58 ± 0.91	0.031^2^
**TG**,** mg/dL**	108 (76, 153)	133 (90, 194)	< 0.001^2^
**BMI kg/m** ^**2**^	25.4 ± 3.1	26.8 ± 3.6	< 0.001^2^
**height**,** kg**	168 ± 8	168 ± 8	0.891^2^
**HbA1**,** %**	5.28 ± 0.32	5.56 ± 0.34	< 0.001^2^
**FPG**,** mmol/L**	5.37 ± 0.36	5.64 ± 0.32	< 0.001^2^
**HBP**			0.281^3^
no	1,874 (86.1%)	165 (83.3%)	
yes	302 (13.9%)	33 (16.7%)	
**TyG**	8.56 ± 0.54	8.79 ± 0.58	< 0.001^2^
**TyG-WC**	733 ± 87	790 ± 95	< 0.001^2^
**TyG-BMI**	218 ± 32	235 ± 36	< 0.001^2^
**TyG-WHtR**	4.37 ± 0.50	4.71 ± 0.56	< 0.001^2^

^1^Mean ± SD; n (%)

^2^Welch two-sample t-test

^3^Pearson’s chi-squared test

Abbreviations: ALT, alanine aminotransferase; AST, aspartate transaminase; BMI, body mass index; DM, diabetes mellitus; FPG, fasting plasma glucose; HbA1c, hemoglobin a1c; HBP, high blood pressure; HDL-C, high-density lipoprotein cholesterol; GGT, gamma-glutamyl transferase; TC, total cholesterol; TG, triglyceride; WC, waist circumference; WHtR, waist-to-height ratio

### Relationships of TyG and modified indices with incident DM among NAFLD patients

**Fig. 1:**
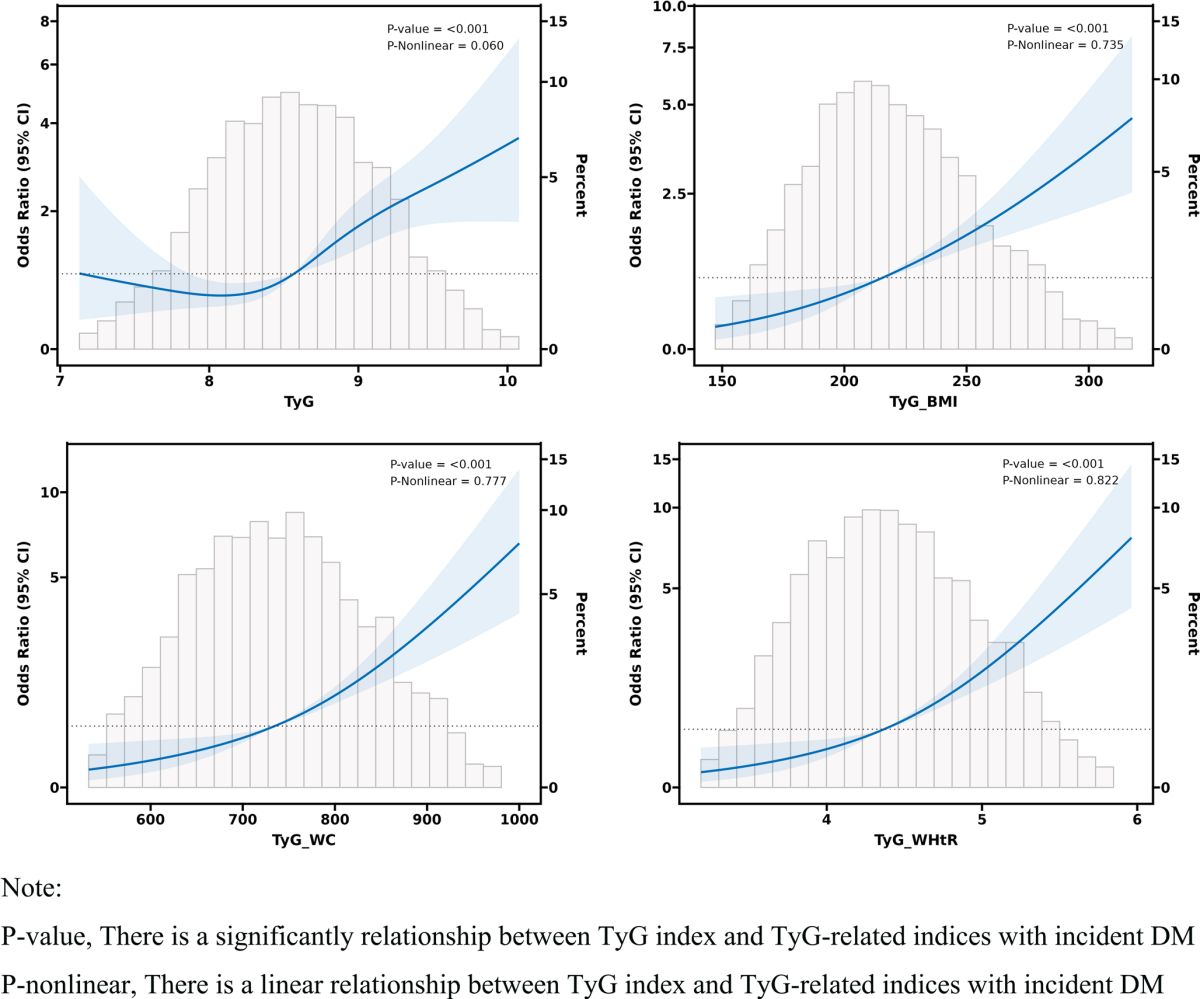
Dose-responsive relationship of the TyG index and modified indices with the risk of incident diabetes mellitus among NAFLD patients

Subsequently, we proceeded to examine the relationship between the TyG index and modified indices with the presence of incident DM using multiple logistic regression analysis (Table 2). A significant positive association was identified between the TyG index and incident DM (OR = 2.19, 95% CI: 1.67–2.87, *P* < 0.001). This association remained robust after adjustment in both the minimal (OR = 1.93, 95% CI: 1.44–2.58, *P* < 0.001) and comprehensive (OR = 1.45, 95% CI: 1.23–1.72, *P* < 0.001) regression models. Furthermore, a sensitivity analysis was conducted, which involved categorizing the TyG index from a continuous to a categorical variable, to validate these findings. In the fully adjusted model, participants in the high group of the TyG index had an adjusted OR of 2.39 (95% CI: 1.56–3.66, *P* < 0.001). A significant positive association was also identified between the modified indices and incident DM. This association remained robust even after adjustments in the comprehensive regression models. Each SD increment corresponded to ORs with 95% CIs of 1.57 (1.33–1.85, *P* < 0.001), 1.81 (1.53–2.14, *P* < 0.001), and 1.75 (1.48–2.06, *P* < 0.001) for TyG-BMI, TyG-WC, and TyG-WHtR, respectively. Furthermore, a sensitivity analysis was conducted to categorize the modified indices from continuous to categorical variables, thereby validating these findings. In the fully adjusted model, participants in the high group of TyG-BMI, TyG-WC, and TyG-WHtR had adjusted ORs of 2.56 (high group: OR = 2.56, 95% CI: 1.66–3.96, *P* < 0.001), 3.21 (high group: OR = 3.21, 95% CI: 2.06-5.00, *P* < 0.001), and 3.07 (high group: OR = 3.07, 95% CI: 1.98–4.77, *P* < 0.001). Additionally, a linear relationship was observed between the TyG index and TyG-related indices with incident DM, as depicted in Fig. 1. To evaluate potential unmeasured confounding factors, we computed E-values. For the TyG index, TyG-BMI, TyG-WC, and TyG-WHtR, the E-values were 2.26, 2.52, 3.02, and 2.90, respectively, demonstrating their stability.

**Table 2:** Associations of TyG index and modified indices with incident diabetes mellitus among NAFLD patients

Variable	Unadjusted			Model 1			Model 2	
	OR (95%CI)	*P* value		OR (95%CI)	*P* value		OR (95%CI)	*P* value
TyG								
Continuous (per SD)	2.19(1.67–2.87)	< 0.001		1.93(1.44–2.58)	< 0.001		1.45(1.23–1.72)	< 0.001
Categorized								
Low	Ref			Ref			Ref	
Middle	1.40(0.92–2.13)	0.117		1.24(0.80–1.91)	0.341		1.24(0.79–1.92)	0.347
High	2.80(1.92–4.10)	< 0.001		2.33(1.56–3.49)	< 0.001		2.39(1.56–3.66)	< 0.001
TyG-BMI								
Continuous (per SD)	1.64(1.44–1.88)	< 0.001		1.59(1.36–1.86)	< 0.001		1.57(1.33–1.85)	< 0.001
Categorized								
Low	Ref			Ref			Ref	
Middle	1.71(1.11–2.63)	0.015		1.58(1.01–2.47)	0.043		1.58(1.00-2.47)	0.048
High	3.30(2.22–4.91)	< 0.001		2.72(1.79–4.13)	< 0.001		2.56(1.66–3.96)	< 0.001
TyG-WC								
Continuous (per SD)	1.85(1.60–2.13)	< 0.001		1.81(1.54–2.12)	< 0.001		1.81(1.53–2.14)	< 0.001
Categorized								
Low	Ref			Ref			Ref	
Middle	1.47(0.94–2.30)	0.092		1.38(0.87–2.21)	0.172		1.36(0.85–2.18)	0.203
High	3.78(2.54–5.62)	< 0.001		3.36(2.20–5.15)	< 0.001		3.21(2.06-5.00)	< 0.001
TyG-WHtR								
Continuous (per SD)	1.88(1.63–2.17)	< 0.001		1.75(1.50–2.04)	< 0.001		1.75(1.48–2.06)	< 0.001
Categorized								
Low	Ref			Ref			Ref	
Middle	1.53(0.97–2.42)	0.068		1.37(0.86–2.20)	0.187		1.36(0.84–2.19)	0.208
High	4.15(2.77–6.22)	< 0.001		3.24(2.13–4.95)	< 0.001		3.07(1.98–4.77)	< 0.001

Model1 adjust for sex, age, HbA1c, smoking status, and HBP

Model2 adjust for sex, age, HbA1c, smoking status, HBP, habit of exercise, ALT, AST, GGT, and total cholesterol

Abbreviations: BMI, body mass index; CI, confidence interval; HBP, high blood pressure; HbA1c, hemoglobin a1c; OR, odds ratio; Ref, reference; SD, standard deviation; TyG, triglyceride-glucose index; WC: waist circumference; WHtR: waist-to-height ratio

### Predictive capacity comparison

**Fig. 2:**
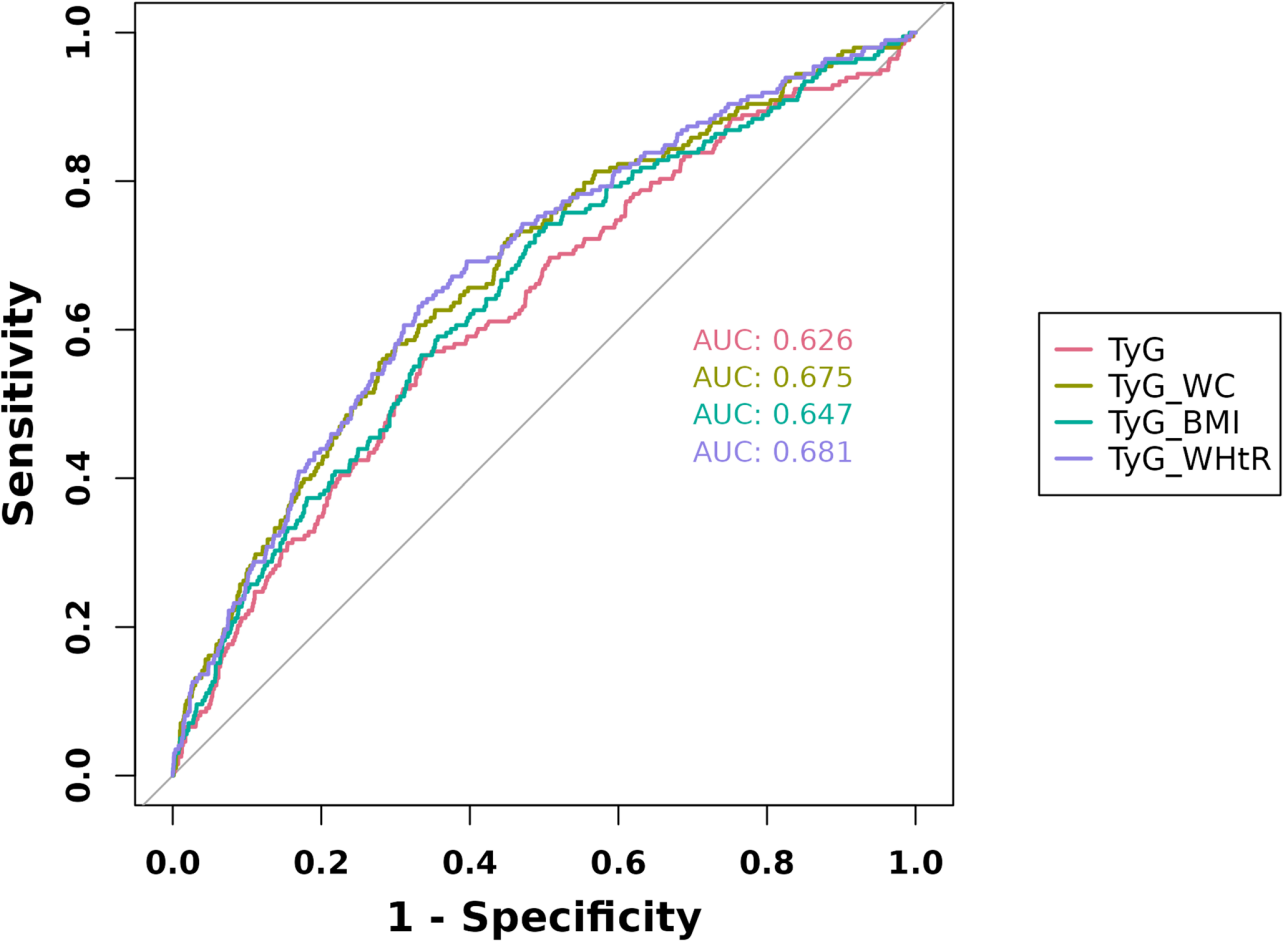
Predictive capacity of TyG index and modified indices for **incident** diabetes mellitus among NAFLD patients

The evaluation of the receiver operating characteristic area under the curve (ROC AUC) for TyG, TyG-BMI, TyG-WC, and TyG-WHtR, pertinent to the development of incident DM, yielded the following scores: TyG achieved 0.626 (95% CI: 0.584–0.668), followed by TyG-BMI at 0.647( (95% CI: 0.635–0.715), TyG-WC at 0.675 (95% CI: 0.607–0.688), and TyG-WHtR at 0.681 (95% CI: 0.641–0.720). Obviously, the TyG-related indices demonstrate superior predictive performance compared to the TyG index alone, with the TyG-WHtR index showing the highest AUC at 0.681 (Fig. 2). These findings highlight the usefulness of these indices in predicting the risk of incident DM, with indices related to the TyG index demonstrating particularly promising discriminative ability in this regard.

### Subgroup analysis

To evaluate changes and interactions, we performed a detailed analysis of the impact of the TyG and modified TyG indices (per standard deviation) on the incidence of DM within subgroups defined by age (< 45 or ≥ 45 years), sex (female or male), hypertension (HBP: yes or no), exercise habits (habit of exercise: yes or no), and smoking status (never, past, or current) (Table 3). Notably, the association was observed across all subgroups. Additionally, no interactions were identified in the subgroup analyses.

**Table 3:** Subgroup analyses of TyG index and modified indices with incident diabetes mellitus among NAFLD patients

Subgroup	TyG			TyG-BMI			TyG-WC			TyG-WHtR		
	OR (95%CI)	*P* value	*P* for interaction	OR (95%CI)	*P* value	*P* for interaction	OR (95%CI)	*P* value	*P* for interaction	OR (95%CI)	*P* value	*P* for interaction
**Age**			0.839			0.216			0.798			0.629
<45	2.06(1.40–3.03)	< 0.001		3.27(1.96–5.46)	< 0.001		2.98(1.81–4.91)	< 0.001		3.25(2.00-5.28)	< 0.001	
≥ 45	2.12(1.54–2.92)	< 0.001		2.02(1.30–3.14)	0.002		2.52(1.60–3.98)	< 0.001		2.56(1.60–4.10)	< 0.001	
**Sex**			0.813			0.377			0.371			0.316
male	2.30(1.25–4.22)	0.007		1.94(0.99–3.78)	0.052		2.18(1.12–4.24)	0.022		2.06(1.02–4.13)	0.042	
female	2.12(1.54–2.92)	< 0.001		2.70(1.84–3.97)	< 0.001		3.21(2.13–4.82)	< 0.001		3.19(2.17–4.69)	< 0.001	
**HBP**			0.716			0.052			0.173			0.322
yes	1.92(0.98–3.76)	0.058		1.19(0.52–2.74)	0.677		1.66(0.68–4.06)	0.267		2.01(0.79–5.11)	0.142	
no	2.08(1.52–2.83)	< 0.001		2.79(1.95-4.00)	< 0.001		2.95(2.05–4.24)	< 0.001		3.07(2.14–4.41)	< 0.001	
**Habit of exercise**			0.861			0.582			0.433			0.764
yes	2.04(0.83–4.97)	0.119		2.14(0.77–5.96)	0.144		1.82(0.67–4.95)	0.241		2.86(2.00-4.07)	< 0.001	
no	2.07(1.54–2.79)	< 0.001		2.58(1.82–3.66)	< 0.001		2.86(1.99–4.10)	< 0.001		3.54(1.20-10.45)	0.022	
**Smoking status**			0.762			0.321			0.636			0.704
Never	1.87(1.24–2.83)	0.003		1.70(1.39–2.08)	< 0.001		1.90(1.53–2.35)	< 0.001		1.93(1.57–2.37)	< 0.001	
Past	2.38(1.40–4.05)	0.001		1.28(0.93–1.76)	0.127		1.69(1.22–2.34)	0.002		1.70(1.22–2.35)	0.001	
Current	1.92(1.26–2.92)	0.002		1.60(1.31–1.95)	< 0.001		1.65(1.33–2.04)	< 0.001		1.73(1.40–2.13)	< 0.001	

Abbreviations: BMI, body mass index; CI, confidence interval; HBP, high blood pressure; OR, odds ratio; TyG, triglyceri de-glucose index; WC: waist circumference; WHtR: waist-to-height ratio

## Discussion

This study aimed to investigate the use of TyG-related indices as an early screening tool for incident DM among patients with NAFLD. The results suggest that TyG, TyG-BMI, TyG-WC, and TyG-WHtR are positively associated with incident DM in patients with NAFLD. The study found that these three modified parameters are more suitable for assessing the occurrence of incident DM and monitoring disease progression in NAFLD patients, which could be a valuable tool for detecting and managing this disease.

The TyG index, calculated using fasting triglyceride and glucose levels, serves as a surrogate marker for insulin resistance (IR). Previous research has confirmed that the TyG index can be used as a surrogate for identifying IR, comparing it to the hyperinsulinemic-euglycemic glucose clamp test, which is the gold standard for assessing IR [19]. This methodological approach highlights the applicability of the TyG index in clinical settings and offers a practical alternative to more invasive or resource-intensive diagnostic procedures. Hyperlipidemia and inadequate glycemic control frequently coexist in patients with NAFLD. DM can result in hepatic lipid accumulation, which in turn triggers hepatocytes to release inflammatory cytokines, such as tumor necrosis factor-alpha (TNF-α) and interleukin-6 [20]. The accumulation of inflammatory mediators further exacerbates the systemic inflammatory response, leading to increased infiltration of cytokines into the liver. This damages insulin target cells and aggravates insulin resistance within the organism [21]. Persistent inflammation triggers hepatic stellate cells to produce collagen, accelerating the progression of liver fibrosis, cirrhosis, and potentially hepatocellular carcinoma, which significantly increases the risk of mortality [22]. This relationship forms a harmful cycle, resulting in patients with glucose metabolic disorders and poor liver function [20]. In a 2015 study conducted in the UK, which utilized data from liver biopsies, it was discovered that among patients initially diagnosed with steatosis without hepatocellular damage, the development of liver fibrosis was significantly associated with DM. Specifically, 80% of patients with progressive fibrosis were diabetic at the time of follow-up biopsies, compared to only 25% of patients without progressive fibrosis [23]. This finding underscores a strong correlation between elevated blood glucose levels and the progression of NAFLD. These results emphasize the potential role of hyperglycemia as a contributing factor to the progression of NAFLD pathology. Therefore, the identification of a simple and non-invasive marker to predict the onset of DM in patients with NAFLD holds significant clinical importance. Prior research studies have revealed elevated TyG levels to be a distinct predictor of DM [24–26], including gestational DM [27]. A prospective study pointed out an increasing relationship between TyG index and new-onset DM [28]. Similarly, another study by Simental-Mendía et al. found that higher TyG index values were associated with an increased risk of developing DM in a cohort of Mexican individuals [29]. Furthermore, our findings align with those previously mentioned. Additionally, prior research has shown a non-linear relationship between these factors. In contrast, our investigation uncovered a positive, linear correlation between the TyG index and DM risk. This is due to the fact that our study population consisted of individuals with NAFLD, which is typically characterized by elevated TyG indices. This is consistent with earlier research suggesting that a high TyG index is linked to an increased risk of DM across various racial groups [30]. Another factor could be that individuals with NAFLD exhibit unique differences in body composition and metabolic rates compared to other groups, often presenting with reduced muscle mass and slower metabolic activity [31]. In summary, the TyG index is a promising and reliable predictor of incident DM in the context of NAFLD and is thus appropriate for widespread clinical application.

Aside from the TyG index, a few past research studies have also unveiled a connection between modified TyG indices and the incidence of DM or Metabolic syndrome (MetS) [32, 33]. A population-based cohort study indicated a causal link between DM incidence and TyG-related indicators, even after accounting for various confounding elements [34]. Significantly, our study observed a marked increase in DM cases associated with the modified TyG indices, which aligns with previously discussed evidence. Furthermore, the research assessed the predictive capabilities of the TyG index against other TyG-related indicators. It was found that the predictive power for incident DM was stronger across all TyG-related indices, specifically TyG-WHtR, TyG-BMI, and TyG-WC, surpassing that of the TyG index alone. Among these, the TyG-WHtR index emerged as potentially the most effective predictor.

Nonetheless, disagreements persist regarding the predictive significance of the TyG index and its associated parameters. In research conducted among the Chinese senior population, it was concluded that the TyG index exhibited superior predictive capacity compared to other TyG-related indicators [35]. Conversely, in the Korean population, TyG-BMI and TyG-WC demonstrated stronger predictive abilities for DM risk than the TyG index [36]. Meanwhile, another study revealed a positive and linear correlation between parameters associated with TyG and the incidence of DM. TyG-WHtR was identified as a significant predictor for the risk of developing DM when forecasting new-onset DM [37], consistent with our study’s findings. The disparity in the aforementioned results may stem from differences in study populations and sample sizes. Additionally, the confounding factors adjusted in previous studies varied, indicating a need for further investigation into this relationship in future research, which should encompass a broader range of races.

The study offers a significant advantage by providing directive value for healthcare professionals, enabling them to identify high-risk populations and predict DM incidence within the context of NAFLD. Furthermore, our research considers a broader range of confounding variables and incorporates E-value sensitivity analysis, which sets it apart from previous studies. This approach enhances the robustness of our findings and minimizes the influence of unmeasured confounders. However, several limitations were inherent in this investigation. Firstly, there was no distinction between type 1 and type 2 DM, suggesting a potential avenue for future studies to concentrate specifically on one diabetes subtype. Secondly, the applicability of the outcomes to different ethnic populations remains unestablished, given that the data originated solely from Japanese participants. Thirdly, as the information is derived from prior published studies, the comprehension of procedural specifics during medical consultations, such as blood pressure measurements, remains incomplete. Lastly, all variables exhibited profound significance, which might be attributed to an unadjusted sample size. However, the large sample base used in the data analysis bolsters the reliability of the findings despite this limitation.

In conclusion, the TyG-related indices were significantly associated with new-onset DM among patients with NAFLD. These three modified TyG indices may also be more useful than the original TyG index for the early detection of disease progression in this population. Future research should focus on validating these findings in larger cohorts and exploring the potential clinical implications of these parameters in the management of NAFLD patients at risk of new-onset DM.

## Data Availability

The original contributions presented in the study are included in the article, further inquiries can be directed to the corresponding author/s.

## Abbreviations

ALT: Alanine aminotransferase
AST: Aspartate transaminase
AUC: Area under curve
BMI: Body mass index
CI: Confidence interval
DBP: Diastolic blood pressure
DM: Diabetes mellitus
FPG: Fasting plasma glucose
GGT: Gamma-glutamyl transferase
HbA1c: Hemoglobin a1c
HBP: High blood pressure
HDL-C: High-density lipoprotein cholesterol
IQR: Interquartile ranges
IR: Insulin resistance
MetS: Metabolic syndrome
NAFLD: Non-alcoholic fatty liver disease
NAGALA: NAFLD in the Gifu Area, Longitudinal Analysis
OR: Odds ratio
ROC: Receiver operating characteristic
SBP: Systolic blood pressure
SD: Standard deviation
TC: Total cholesterol
TG: Triglyceride
TNF-α: Tumor necrosis factor-alpha
TyG: Triglyceride glucose
WC: Waist circumference
WHtR: Waist-to-height ratio
